# All in the Family: Child and Adolescent Weight Loss Surgery in the Context of Parental Weight Loss Surgery

**DOI:** 10.3390/children8110990

**Published:** 2021-11-01

**Authors:** Eleanor R. Mackey, Megan M. York, Evan P. Nadler

**Affiliations:** 1 Children’s National Hospital, Washington, DC 20010, USA; myork@childrensnational.org (M.M.Y.); enadler@childrensnational.org (E.P.N.); 2 School of Medicine and Health Sciences, George Washington University, Washington, DC 20037, USA

**Keywords:** bariatric surgery, children, adolescents, parents, psychosocial factors

## Abstract

Background: Bariatric surgery is the most effective current treatment option for patients with severe obesity. More children and adolescents are having surgery, many whose parents have also had surgery. The current study examines whether parental surgery status moderates the association between perceived social support, emotional eating, food addiction and weight loss following surgery, with those whose parents have had surgery evidencing a stronger relationship between the psychosocial factors and weight loss as compared to their peers. Methods: Participants were 228 children and adolescents undergoing sleeve gastrectomy between 2014 and 2019 at one institution. Children and adolescents completed self-report measures of perceived family social support, emotional eating, and food addiction at their pre-surgical psychological evaluation. Change in body mass index (BMI) from pre-surgery to 3, 6, and 12 months post-surgery was assessed at follow-up clinic visits. Parents reported their surgical status as having had surgery or not. Results: There were no differences in perceived family support, emotional eating, or food addiction symptoms between those whose parents had bariatric surgery and those whose parents did not. There were some moderating effects of parent surgery status on the relationship between social support, emotional eating/food addiction, and weight loss following surgery. Specifically, at 3 months post-surgery, higher change in BMI was associated with lower perceived family support only in those whose parents had not had surgery. More pre-surgical food addiction symptoms were associated with greater weight loss at 3 months for those whose parents had not had surgery, whereas this finding was true only for those whose parents had surgery at 12 months post-surgery. Conclusions: Children and adolescents whose parents have had bariatric surgery may have unique associations of psychosocial factors and weight loss. More research is needed to determine mechanisms of these relationships.

## 1. Introduction

The obesity epidemic in the United States affects both adults and children, with a remarkable projection that by 2030, more than 75% of adults will suffer from obesity [1]. Rates of obesity amongst children and adolescents are also high, with rates of severe obesity (defined as body mass index/BMI kg/m^2^ ≥ 120% of the 95th percentile) as high as 4.5% amongst adolescents [2]. Severe obesity in childhood and adolescence is associated with higher rates of obesity and significant cardiovascular risk factors into adulthood [3].

### 1.1. Bariatric Surgery

Severe obesity has demonstrated resistance to the standard first-line treatments of lifestyle changes and medication [3]. However, bariatric surgery has been shown to be an effective treatment in both adults [4] and children and adolescents [5]. Given the influence of genes and shared environment on the development of obesity, ref. [6] children of parents with severe obesity are likely to suffer from severe obesity themselves [7].

As rates of bariatric surgery increase in general, [8] there are therefore greater numbers of children and adolescents undergoing bariatric surgery whose parents have also received surgery. Recent research has examined the impact of parental bariatric surgery on children but findings in this population have been mixed. For example, one study found that children of parents who have undergone bariatric surgery report less exercise and more disordered eating behaviors as compared to children whose parent had not undergone surgery [9]. In contrast, other studies found no changes in food choices in the home nor a significant change in BMI in children after a parent’s surgery [10]. Given that this is a growing population, more research is needed not only on the impact of parental surgery on all children, and specifically those youth who themselves undergo bariatric surgery.

### 1.2. Social Support and Surgery

Parents provide important support for their children, and this support is particularly important for those children and adolescents who have chronic health conditions, such as obesity [11]. Although the role of social support in the context of bariatric surgery is understudied, limited research available indicates that perceived social support may be associated with eating and exercise in adolescents undergoing bariatric surgery [12]. With regard to children of parents who have had bariatric surgery, research has noted that there are associations with poorer family functioning and less support for healthful eating and exercise for those with children who also suffer from obesity [13]. Together, these findings indicate that the potential buffer of social support from families in bariatric surgery for children and adolescents overall and that family functioning may be poorer in families with children and adolescents with obesity whose parents have had bariatric surgery themselves.

### 1.3. Family Transmission of Eating Behaviors

Another set of behaviors that may be transmitted through families and therefore may be more likely to impact children and adolescents undergoing bariatric surgery whose parents have also undergone surgery is emotional eating and food addiction. Existing literature suggests that the children who have overweight are more likely to develop emotional eating if their parents engage in emotional eating [14]. Similarly, food addiction behaviors appear to be greater in youth whose parents also experience symptoms of food addiction, suggesting the potential for intergenerational transmission [15]. Since rates of food addiction may be higher in those seeking bariatric surgery as compared to other populations, [16] it is possible that there are also higher rates of food addiction in children of parents who have had bariatric surgery than their peers whose parents have not had bariatric surgery.

### 1.4. Current Study

As more children and adolescents of parents who have had bariatric surgery seek surgery themselves, it is important to understand the unique context of these youth and whether there is an association of parental surgery and pre-surgical behaviors and post-surgical weight loss in adolescents. Given the potential importance of social support and emotional eating and food addiction behaviors that may be influenced by having parents who have undergone surgery, it is important to explore these relationships further. Therefore, the current study aims to evaluate whether there is a difference in perceived pre-surgical family support, emotional eating, and food addiction in children and adolescents whose parents have had bariatric surgery in comparison with those whose parents have not had surgery. We hypothesize that children of parents who have had surgery will report less pre-surgical perceived family support, consistent with the finding of Pratt and colleagues (2020), [13] than peers whose parents have not had surgery. We also hypothesize that there will be more emotional eating and food addiction reported by those children and adolescents whose parents have had bariatric surgery. Finally, the current study examines whether parental surgery status moderates the association between perceived social support, emotional eating, food addiction and weight loss following surgery, with those whose parents have had surgery evidencing a stronger relationship between the psychosocial factors and weight loss as compared to their peers.

## 2. Materials and Methods

### 2.1. Participants

All children and adolescents who underwent surgery (all received sleeve gastrectomy) at our single institution from 2014 to 2019 were included in the initial evaluation, given that the self-report measures assessing constructs of interest for the current study were introduced to the pre-operative evaluation in 2014. Only those who completed the pre-operative questionnaires regarding perceived social support, emotional eating, or food addiction (*n* = 228) were included in the final analyses (see Table 1 for sample size for each measure).

### 2.2. Procedures

Participants completed self-report measures during their pre-surgical psychological functioning evaluation that occurred between 1 and 4 months prior to surgery. All weight and weight loss measures were taken at regular clinic visits with the surgeon or the medical weight management clinic prior to and following surgery. All methods were approved by the Institutional Review Board. Participants completed an informed consent process to allow their clinical data to be utilized for research purposes. Participants were not compensated for their participation as all data were completed as part of regular clinical procedures rather than for research purposes.

### 2.3. Measures

#### 2.3.1. Perceived Social Support

The Multidimensional Scale of Perceived Social Support, [17] Family subscale, was used to assess adolescents’ perception of general (not surgery-specific) support they receive from their families. The Family subscale is comprised of four items rated on a scale of 1 (very strongly disagree) to 7 (very strongly agree) with higher scores indicating greater perceived social support, assessing how much family is perceived to listen to problems/concerns, make decisions, and provide help and emotional support. The subscale is calculated using the mean of the four items in the scale and has established reliability and validity [18] and good internal consistency in the current sample (α = 0.92).

#### 2.3.2. Food Addiction

The Yale food addiction scale for Children was used as an indicator of symptoms of food addiction. This is a 25-item scale with established reliability and validity [19]. For the current study, the total symptom score was used as an indicator of level of food addiction reported at the pre-surgical evaluation. This measure demonstrates good internal consistency in the current sample (α = 0.89).

#### 2.3.3. Emotional Eating

To assess emotional eating, adolescents completed the Emotional Eating Scale for Children, a 27-item measure with established reliability and validity [20] rating their level of desire to eat under specific emotional conditions on a scale of 1 (no desire to eat) to 5 (very strong desire to eat). The measure is comprised of three subscales of anxiety/anger/frustration, depression, and feeling unsettled. To minimize increased risk of type 1 error, the mean of all three subscales was computed for the current analyses. This measure demonstrates good internal consistency in the current sample (α = 0.95).

#### 2.3.4. Weight Loss

To estimate weight loss, change in body mass index (BMI) from pre-operative at 3, 6, and 12 months post-surgery was calculated. Weight and height were gathered using trained clinical providers using stadiometers and calibrated scales as part of regular clinic visits. Higher (positive) values indicate greater weight loss. There were 15 participants who had follow-up data at 3 and 12 months but were missing the intervening month data points. For each of these cases, the average of the two changes in BMI obtained at 3 and 12 month post-operative time points was used to calculate 6 month weight loss. Pre-operative BMI (kg/m^2^) was also used as a control variable, given that it is associated with post-operative weight loss [21].

#### 2.3.5. Data Analytic Plan

Descriptive data were analyzed to characterize the sample. To address whether there was a difference in perceived social support, emotional eating, and food addiction, independent-samples *t*-tests were conducted between those whose parents had surgery and those whose parents had not. Distribution of the variables was normal and the variances were equal (based on Levene’s test). To evaluate whether parents having surgery moderated the association between emotional eating, food addiction, and weight loss, moderation analyses using the Preacher and Hayes process macro version 3.5 were conducted [22]. Outcome measures were 3, 6, and 12 month change in BMI, moderated by whether a parent had surgery or not, and predictors were family support, food addiction, and emotional eating, with pre-operative BMI included as a covariate. Analyses were completed using IBM SPSS Version 26 for Macintosh.

## 3. Results

Participants were 228 children and adolescents aged 10–24 (Mean age = 17.2 years, SD = 2.2 years). The majority were female (71%). Participants represented diverse backgrounds with 53% Black, 27% White, 15% Latinx, and 5% Other. Pre-operative BMI ranged from 35 to 87 (mean = 49.5, SD = 8.7). Forty-four patients had parents who had a history of bariatric surgery and 184 did not have a parent with a history of bariatric surgery. Table 1 includes descriptive information for the total sample and by parent surgery group, as well as the sample size for each of the variables. Overall, participants reported a moderate amount of perceived family support, minimal food addiction symptoms, and low desire to eat under various emotional states (see Table 1).

Independent-samples *t*-tests revealed no significant differences in perceived family support prior to surgery for those whose parents had bariatric surgery as compared to those who did not (t(226) = 0.04, *p* > 0.05). Similarly, total food addiction symptoms were not significantly different between groups (t(179) = 0.76, *p* > 0.05). There was no difference in reported emotional eating between the two groups (t(179) = 0.98, *p* > 0.05).

**Figure 1:**
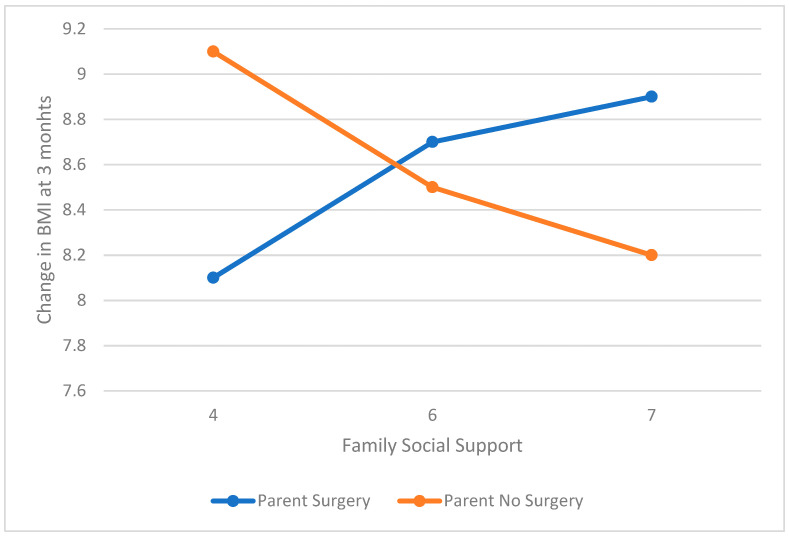
Pre-surgical perceived family social support and 3 month change in BMI (kg/m^2^) moderated by parent surgery.

The models evaluating the association between family social support and 3, 6, and 12 month change in BMI moderated by parental surgery were significant. At 3 months post-surgery, there was a trend for a moderating influence of parental weight loss surgery (interaction coefficient = 0.58, t = 1.73, *p* = 0.09, SE = 0.33, 95% CI = −0.08–1.24). Specifically, the relationship between family support and weight loss at 3 months was only significant for those whose parents had not had surgery (coefficient = −0.30, t = −2.28, *p* = 0.02, SE = 0.13, 95% CI= −0.57–−0.04) as opposed to those whose parents had surgery (coefficient = 0.28, t = 0.90, *p* > 0.05, SE = 0.31, 95% CI = −0.33–0.89). Notably, higher social support in the non-parental surgery group was associated with lower change in BMI at 3 months (see Figure 1). At 6 and 12 months post-surgery, only pre-operative BMI was associated with weight loss, with no association of family support, family surgery, or an interaction between the two (all *p* > 0.05).

**Figure 2:**
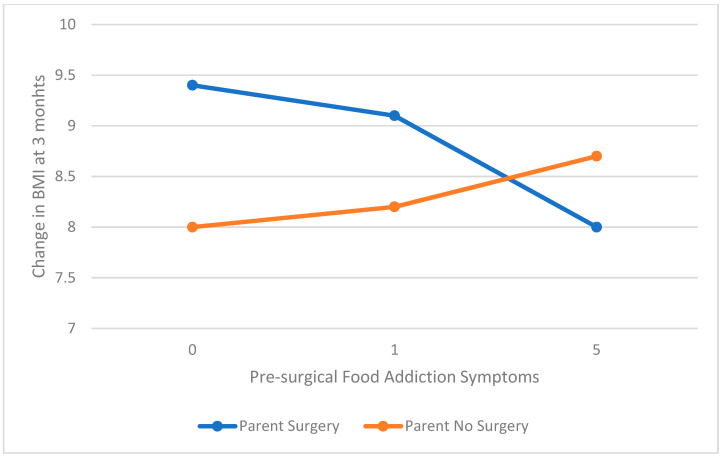
Pre-surgical food addiction and 3 month change in BMI (kg/m^2^) moderated by parent surgery.

**Figure 3:**
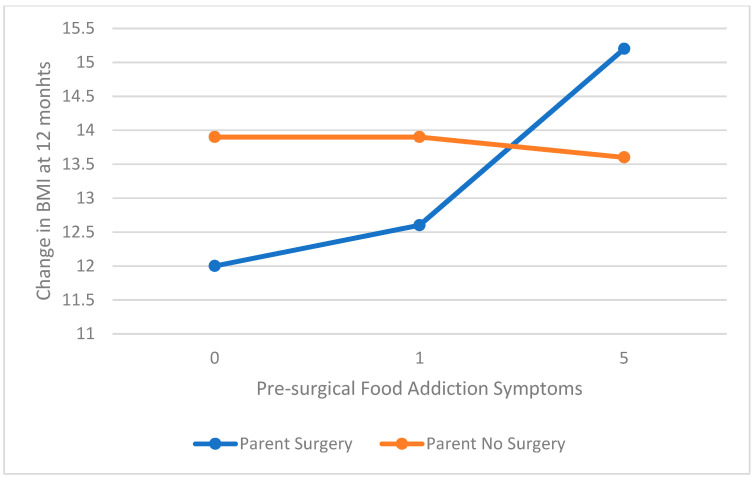
Pre-surgical food addiction and 12 month change in BMI (kg/m^2^) moderated by parent surgery.

The models evaluating the association between food addiction and 3, 6, and 12 month change in BMI moderated by parental surgery were significant. The primary contributor to the model was pre-operative BMI. At 3 months post-surgery, there was a significant moderation of parental surgery on the association of food addiction and change in BMI (interaction coefficient = −0.43, t = −1.98, *p* = 0.05, SE = 0.22, 95% CI = −0.86–0.00). For those whose parents had not had surgery, higher food addiction symptoms pre-surgery was associated with greater change in BMI, whereas the reverse was true for those whose parents had surgery (see Figure 2). At 12 months there was a trend for a moderating effect of parental surgery (interaction coefficient = 0.72, t = −1.73, *p* = 0.09, SE = 0.41, 95% CI = −0.11–1.5). Unlike at 3 months post-surgery, symptoms of food addiction were not associated with weight loss for those whose parents did not have surgery but increase in food addiction reported prior to surgery was associated with greater change in BMI at 12 months for those whose parents had surgery (see Figure 3). At 6 months, there were no significant interactions or contributions of parental surgery or food addiction to change in BMI (all *p* > 0.05).

The models evaluating the association between emotional eating and 3, 6, and 12 month change in BMI moderated by parental surgery were significant. The primary contributor to the model was pre-operative BMI, with no significant contribution of parental surgery or emotional eating (all *p* > 0.05).

## 4. Discussion

The current study evaluated whether children and adolescents having bariatric surgery whose parents also had bariatric surgery differed in family support and disordered eating prior to surgery from peers who did not have a parent with a history of bariatric surgery. This study also evaluated whether having a parent with a history of bariatric surgery moderated the association between these psychosocial factors and weight loss following surgery. Consistent with previous literature, the primary contributor to weight loss following surgery was pre-operative BMI [21]. Contrary to hypotheses, children and adolescents whose parents had surgery did not have lower levels of perceived family support prior to surgery, nor did they have increased food addiction or emotional eating symptoms.

Having a parent who had bariatric surgery did, however, demonstrate a trend towards moderating the association between family support and weight loss at 3 months post-surgery, but not in later post-surgical periods. Contrary to hypotheses, greater perceived family support prior to surgery was associated with less weight loss for those whose parents had not had surgery, whereas there was a trend for greater weight loss with higher perceived support for those whose parents had surgery. This, perhaps, can be interpreted as indicating that in that transition period through meal replacement shakes to soft food, having a parent who has been through this process and who is perceived as supportive is particularly beneficial, whereas family support from those who have not lived the experience from surgery themselves may be perceived in a negative way in this sensitive period following surgery. Additional research employing measures following surgery, measures that are specific to post-surgical support, as well as qualitative research to evaluate these processes in greater depth are needed to better understand these findings. The current study also did not evaluate factors such as parental weight loss in those who had surgery and how this might affect support and/or adolescent outcomes.

Interestingly, food addiction symptoms were related to weight loss following surgery, moderated by parent surgery status differently at 3 months post-surgery than 12 months post-surgery. At 3 months post-surgery, there was a trend for higher pre-surgical food addiction symptoms to be associated with more weight loss for those whose parents had not had surgery. However, at 12 months, there was no association of food addiction with weight loss for those whose parents had not had surgery. Moreover, for those whose parents had surgery, higher food addiction symptoms pre-surgery was associated with greater change in BMI at 12 months post-surgery. These data need to be replicated with a larger sample size but suggest the possibility that there may be an immediate attenuation of food addiction symptoms post-surgery that may drive higher immediate weight loss following surgery for those whose parents have not had surgery. Alternatively, initial weight loss may be affected by factors other than attenuation of food addiction symptoms in youth with parents who have had surgery, but pre-surgical food addiction symptoms may have a larger contribution to weight loss a year post-surgery. This hypothesis would require research examining changes in food addiction symptoms following surgery [23].

Consistent with existing literature, [24] pre-surgical emotional eating does not appear to be associated with weight loss following surgery, either for children and adolescents whose parents did or did not have surgery. This does not mean that post-surgical emotional eating does not play a role in weight loss and should not be treated, but indicates that high levels of emotional eating prior to surgery may not warrant preventing adolescents from seeking surgery.

The primary limitation of the current study is the small sample size, particularly for those with parents who have had surgery. Because of the discrepancy in sample size between the two groups, it is possible that the group whose parents had not had surgery simply had more power to detect associations between psychosocial factors and weight loss following surgery. Additionally, there is no perfect measure of weight loss following surgery. Change in BMI is an easy to interpret number as it translates to lost BMI points, which is a similar metric to that reported in behavioral weight loss research. However, it is affected by pre-operative BMI, and therefore all analyses accounted for pre-operative BMI. The psychosocial data were all collected prior to surgery and it is very possible that post-operative perceptions and behaviors are most important for weight loss. Finally, there are many other factors which could be included in order to evaluate these relationships including parental success with weight loss and parental emotional eating and food addiction. Future research should use larger samples, as well as self-report in the period following surgery.

## 5. Conclusions

As the number of bariatric surgeries in children and adolescents, as well as adults continues to grow, more children and adolescents of parents who have had surgery will have surgery themselves. Understanding the unique features of this population with regard to where support may be needed, as well as learning from benefits they may garner from having family members with bariatric surgery experience, can help inform care for all children and adolescents with severe obesity seeking bariatric surgery. The current study indicates the need for more research with a focus on the intersectionality of patient identities and psychosocial factors as potentially critical for evaluating the association of demographic and psychosocial factors on weight loss outcomes.

## Figures and Tables

**Table 1:** Descriptive statistics.

	Overall					Parent Surgery				Parent No Surgery			
	%	M	SD	Range	N	%	M	SD	N	%	M	SD	N
Age (years)		17.2	2.2	10–24			16.4	2.3	44		17.2	2.4	184
Sex (% female)	71				228	64			44	72			184
Pre-operative BMI (kg/m^2^)		49.5	8.7	35–87	228		50.3	11.6	44		49.3	7.8	184
Family social support		5.7	1.5	1–7	228		5.7	1.4	44		5.7	1.5	184
Yale food addiction scale		2.5	2.9	0–15	181		2.1	2.5	35		2.5	3.0	146
Emotional eating scale total		1.8	0.7	1–4.3	181		1.7	0.7	35		1.8	0.7	146
3 months change BMI		8.6	2.6	3–19.5	189		8.6	2.4	34		8.6	2.7	155
6 months change BMI		11.5	2.9	5.5–21.8	155		10.6	2.9	31		11.7	2.9	124
12 months change BMI		13.9	4.9	2.3–36.5	113		14.1	6.8	26		13.8	4.1	87

Independent *t*-tests indicated no significant differences between groups.

## Data Availability

The data presented in this study are available on request from the corresponding author.
